# Curated Collections for Educators: Eight Key Papers about Feedback in Medical Education

**DOI:** 10.7759/cureus.4164

**Published:** 2019-03-01

**Authors:** Sreeja M Natesan, Sara M Krzyzaniak, Christine Stehman, Rebecca Shaw, David Story, Michael Gottlieb

**Affiliations:** 1 Emergency Medicine, Duke University Medical Center, Durham, USA; 2 Emergency Medicine, University of Illinois College of Medicine at Peoria, Peoria, USA; 3 Emergency Medicine, Indiana University School of Medicine, Indianapolis, USA; 4 Emergency Medicine, Gold Coast University Hospital, Queensland, AUS; 5 Emergency Medicine, Wake Forest Baptist Medical Center, Winston-Salem, USA; 6 Emergency Medicine, Rush University Medical Center, Chicago, USA

**Keywords:** feedback, medical education, curated collection, modified delphi method, formative assessment

## Abstract

Feedback is an essential part of learning, growth, and academic success. Junior faculty members are often unfamiliar with the grounding literature that defines feedback. Many times they receive little education on providing and receiving feedback, resulting in unhelpful “feedback” for both learners and program leadership alike. This article aims to summarize eight key papers on feedback, to outline relevant information for emerging clinician educators, and identify ways to use these resources for the faculty development.

In order to generate a list of key papers that describes the importance and significance of feedback, the authors conducted a consensus-building process to identify the top papers. In August and September, 2018, the 2018-2019 Academic Life in Emergency Medicine (ALiEM) Faculty Incubator program discussed the topic of feedback in medical education. A number of papers on the topic was highlighted. This list of papers was further augmented using the suggestions and expertise of guest experts who are leaders in the field of medical education and feedback. The authors also used social media to conduct an open call on Twitter for important papers regarding feedback (utilizing #meded, #Feedback hashtags). Via this process, a list of 88 key papers was identified on the topic of feedback in medical education. After compiling these papers, the authorship group engaged in a modified Delphi approach to build consensus on the top eight papers on feedback. These papers were deemed essential by the authors and have been summarized with respect to their relevance to junior faculty members and to faculty developers.

In this manuscript, we present eight key papers addressing feedback in medical education with discussions and applications for junior faculty members and faculty developers. This list of articles that can serve to help junior clinician educators grow in their ability to give effective feedback and also serve as resources upon which senior faculty can design the faculty development sessions.

## Introduction

Feedback is an essential part of learning, growth, and academic success. It is described as providing specific information of observed performance with respect to a standard, with the intent to improve the learner’s knowledge and performance to reach a common goal [1-4]. Giving effective feedback is seen as a critical link in the teaching-learning interaction. It is considered a critical part of a learner’s education helping them to grow in knowledge acquisition and performance of crucial skills while becoming a competent provider [5-6]. Junior faculty members are often unfamiliar with the grounding literature that defines feedback. Many times, junior faculty struggle as they may receive little education on providing and receiving feedback, resulting in unhelpful “feedback” for both learners and program leadership alike [7-8]. In this article, the authors aim to summarize eight key papers on feedback, to outline relevant information for emerging clinician educators, and to identify ways to use these resources for faculty development. 

## Materials and methods

**Figure 1:**
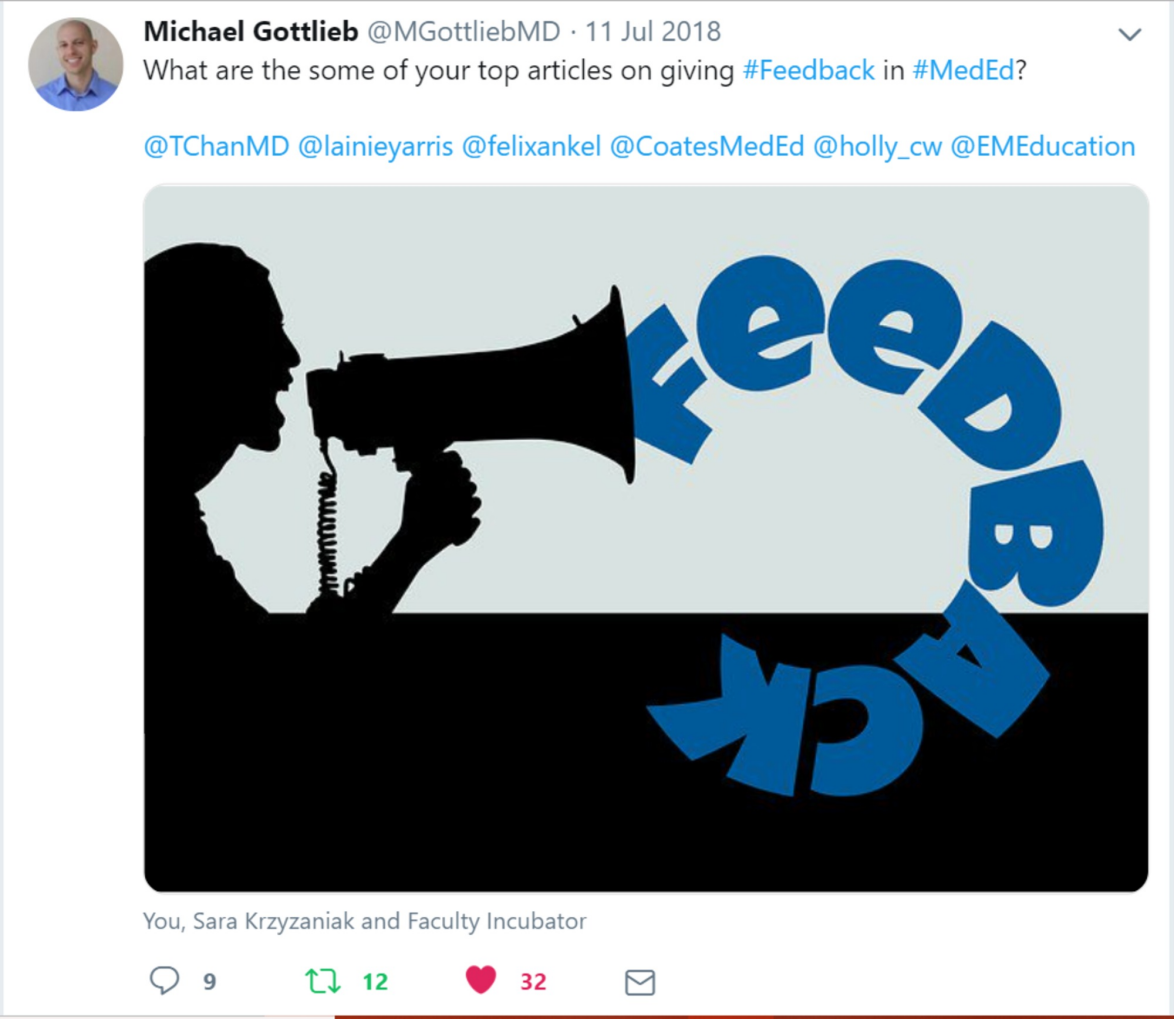
Twitter Call for Feedback Article

The Academic Life in Emergency Medicine (ALiEM) Faculty Incubator is an international professional development community of practice which involves 32 junior faculty members and 20 mentors to share an understanding of medical education and scholarship. During August and September 2018, the junior faculty educators and mentors of the program discussed the topic of feedback using an online discussion forum. Participation in the discussion was required for all of the junior faculty members. Mentors helped to facilitate discussion although participation was not strictly monitored. The titles of articles addressing feedback which were cited, shared, or recommended were compiled into a list. The authors also utilized social media (Twitter) by ‘tweeting’ requests to have participants of the free open access medical education (#FOAMed and #MedEd) online communities of practice provide suggestions for important papers on the topic of feedback [9]. Figure 1 demonstrates an example tweet from the call for articles that was placed via Twitter. Several papers were suggested by more than one modality. 

To ensure the selected papers would be of use to a wide variety of educators throughout their spectrum of their careers, we intentionally involved junior educators in addition to experts [10]. Our consensus group consisted of seven members, including four junior clinician educators (i.e. junior faculty members participating in the Faculty Incubator) as well as three experienced clinician educators (i.e. medical educators, all of whom have published > 10 peer-reviewed publications, or serve as mentors in the ALiEM Faculty Incubator).

A three-round voting method akin to the Delphi methodology was utilized to determine consensus on the top eight papers [11]. All of the articles were read in full by all of this manuscript authors. The first round asked the raters to indicate the importance of each article on a seven-point Likert scale. The Likert scale was anchored at one by the statement "unimportant for junior faculty" and at seven by the statement "essential for junior faculty." In the second round, the participants were provided with the results of the first round including the percent of raters and frequency histogram displaying how each article had been rated. They were then asked to indicate if each article "must be included in the top papers" or "should not be included in the top papers." Participants in this second round could choose as many articles as they deemed appropriate to be included as the “must be included” papers. The third round consisted of the participants being given the results of the second round as a percentage of raters who indicated that each article must be included. They were then asked to select only their top eight papers which should be included in the article because they are the most important. Similar methods were used by the ALiEM Faculty Incubator in prior series [12-23].

## Results

The ALiEM Faculty Incubator discussions, expert recommendations, and social media requests yielded 88 articles. The paper evaluation process resulted in a rank-order listing of these papers in order of perceived relevance as indicated by the results of round three using the Modified Delphi approach. The group decided on using the top eight articles instead of 10 as there was 100% agreement on these. The top eight papers are expanded upon below. 

## Discussion

The following is a list of papers on feedback. These papers were determined to be of interest and relevance to junior faculty members and faculty development officers. Accompanying commentaries are included and are meant to discuss the relevance of these papers to junior faculty members.

*1. *Ende J: Feedback in clinical medical education. JAMA. 1983, 250:777-81. 10.1001/jama.1983.03340060055026 [1]

Summary: Ende's seminal essay was the first of its kind to use the word 'feedback' to describe the practice of providing information to medical learners about their performance in order to guide future performance. He begins with a brief discussion explaining the feedback process to teachers and learners in the medical field and provides a definition of feedback for the clinical realm. In an adjustment from systems and humanities literature, feedback is defined as insight offered to a learner student as to what they did (good and bad) and the consequences of the actions taken. This insight provides a potential disconnect between the learner’s intentions and actual results. The goal of medical training is proficiency in treating patients and feedback provides a non-judgmental method of correcting mistakes, reinforcing appropriate behaviors, and guiding the learner toward competence. Feedback is described in this article as information that is formative and objective in nature. This is in contrast to evaluation which is summative and judgment-based. The two terms should not be used interchangeably. Ende believes there is a paucity of feedback in medical education because of a limited amount of direct observation of the learner, emotional responses of the learner leading to negative effects beyond the intent, and the teacher’s fear of hurt feelings, damaging the student-teacher relationship or leading to negative evaluations from students. He puts forth eight guidelines for delivery of feedback in clinical medicine. While a departure from the “vanishing feedback” that has permeated medical education, incorporating these recommendations into feedback is a challenge only in remembering to utilize them. Ende contests that providing appropriate feedback is necessary, valuable, and not that difficult.

Relevance to Junior Faculty Members: Educating residents and students is a key role of academic faculty members. Being able to provide appropriate, timely, and effective feedback is a required skill for achieving that goal. Most residency programs spend little, if any, time providing direct instruction or education to faculty regarding the provision of learner feedback. Ende’s paper is the building block upon which most subsequent research and recommendations are built. The guidelines offer concrete recommendations for an instructor to follow. Understanding the goals of feedback in the clinical realm, the difference between feedback and evaluation, the risks of inadequate feedback, and the benefits of meaningful feedback provide an excellent starting point for a junior faculty member.

Considerations for Faculty Developers: This paper can be a valuable starting point for faculty developers looking to provide faculty with a strong basis in feedback in medical education. As the seminal article on feedback, it highlights some of the barriers to achieving meaningful feedback, while also providing a roadmap to the early instructor by providing the guidelines for giving effective feedback. This article could be used to build a series of workshops on feedback as it serves as a key article to include in any feedback-giving guide.

*2. *Richardson B: Feedback. Acad Emerg Med. 2008, 11:1283. 10.1197/j.aem.2004.08.036 [23]

Summary: Founded in Ende’s seminal work on feedback, this article aims to provide a solid definition of feedback in the context of student learners in the Emergency Department (ED) while detailing the goals of feedback and consequences of not providing it appropriately [1]. The author then discusses ten “elements of effective feedback” within this context that are grounded in the Accreditation Council for Graduate Medical Education (ACGME)’s five domains of feedback (ie, timeliness, respect/communication, specificity, action plan, and feedback culture). Within these ten elements are themes of addressing the action not the person and making sure to both discuss the problem and the solution. The article continues with a brief discussion of common faculty concerns regarding providing constructive criticism and how to develop faculty who regularly provide learners with appropriate feedback. Finally, the author closes with eight “helpful hints and examples” that extend from the ten elements and are specific to providing feedback in the context of supervising students while providing patient care (particularly in the ED).

Relevance to Junior Faculty Members: Feedback can be challenging to provide. This may be particularly difficult in the ED setting, where are a variety of learners with differing skill and interest levels. This article is a valuable resource for junior faculty as they learn how best to construct feedback to their learners. The examples will help faculty determine which aspects of feedback they need to work on and then focus on each component as needed.

Considerations for Faculty Developers: The author both provides ten elements that will serve as a basis to build faculty development courses around and suggests specifically how to build a feedback course. Faculty developers could use this to design a course or provide this to faculty as a resource.

*3. *Omer AAAA, Abdularhim ME: The criteria of constructive feedback: the feedback that counts . J Health Spec. 2017, 5:45-48. 10.4103/2468-6360.198798 [24]

Summary: This review paper begins by briefly defining feedback and discussing the positive impact of “good” feedback and the negative impact of “feedback done badly”. The authors discuss the concept of “constructive feedback” and its importance for feedback acceptance. The authors then review of the feedback literature to detail both the general conceptual characteristics of constructive feedback. They detail the specific features that are required to construct a solid constructive feedback message that will be heard and acted upon by the feedback recipient.

Relevance to Junior Faculty Members: Educators face many barriers to providing constructive feedback/positive criticism. The authors supply information to help faculty provide effective feedback and build an environment in which feedback will be heard. Junior faculty can use this technology to refine their feedback technique.

Considerations for Faculty Developers: The authors focus on the most challenging aspect of feedback: positive criticism. This article provides faculty with an initial foundation of knowledge of the topic. Faculty developers may use the conceptual characteristics to do an overall needs assessment of their institution’s feedback culture and to generate faculty development courses based on the results. Faculty developers may also choose to focus on the individual faculty, assessing their strengths and weaknesses in each of the 14 components of the feedback message and then developing curricula around the most lacking components.

*4. *Ramani S, Konings K, Ginsburg S, van der Vleuten C: Twelve tips to promote a feedback culture with a growth mind-set: swinging the feedback pendulum from recipes to relationships. Med Teach. 2018,1-7. 10.1080/0142159x.2018.1432850 [25]

Summary: Ramani and colleagues set the stage in this article by discussing the shift in feedback from teacher-oriented to learner-focused. They perform a deep dive into the literature, drawing on several topics and papers that define and explain the reasons for the paradigm shift. The authors provide twelve recommendations for promoting this change by showing a 360-degree interpretation of these tips through four different “lenses” (ie, from the standpoint of the teacher, the learner, the feedback relationship, and the institution). A viewpoint from each of these “lenses” is critical as feedback must be acceptable, meaningful, and supported by all these participants in order to facilitate a learning environment that allows for effective, honest feedback. The tips provided from the teacher aspect include strategies to increase credibility and generate a feedback-accepting climate. The student perspective recommendations function to stimulate a growth mindset within the learner with a goal of increasing receptivity to feedback. Guidelines relating to the feedback relationship include strengthening of the alliance between teacher and learner and emphasizing that involved parties must work together toward a common goal of performance improvement. The institutional role is centered on changing the culture to focus on professional growth and creating an environment where learners and teachers understand that feedback, both positive and negative, is a key element of medical education. The underlying theme of the paper highlights the transition of feedback to a sociocultural process that is focused on the learner’s goals, and the culture change that must occur at the individual, group, and institutional levels to successfully adopt it.

Relevance to Junior Faculty Members: The promotion of relationships and the change to a learner-centered feedback process is of critical importance to junior faculty. The relevance of this article lies in providing the different lenses through which to view the recommendations. It is beneficial to consider not only the teacher’s beliefs, but also the learner’s point of view and role in any feedback interaction. Understanding that the student-teacher relationship can have a dramatic effect on the reception of feedback is also paramount. Methods of enhancing that relationship will only improve the honesty and meaningfulness of the feedback exchanged. Additionally, the participatory design feedback loop that the authors propose provides step-by-step instruction for incorporating a learner-driven plan to assimilate feedback. This article helps create practical tools for the junior learner to incorporate into practice. 

Considerations for Faculty Developers: The articles discusses institutional changes that need to occur in order to successfully implement the strategies put forth in the paper. Faculty developers will be able to design a plan for initiating the culture change that needs to occur at the departmental and institutional levels. In order for this paradigm shift to occur, the environment and culture of feedback need to change, and the tips in the article provide some guidance in that regard. Culture change requires buy-in from the entirety of the parties involved, and in order to get that buy-in, the developers would need to explain reasoning and provide examples and teaching for how this feedback process should happen. Ramani and colleagues provide a reasonable blueprint for invoking the type of change necessary to implement a feedback culture change of this magnitude.

*5. *Kornegay, Joshua G., et al. “Feedback in Medical Education: A Critical Appraisal.” AEM Education and Training, vol. 1, no. 2, 2017, pp. 98-109 [26]

Summary: This article provides a critical appraisal of the literature on feedback with a focus on the top 20 papers with respect to their methodology. The authors performed a systematic review and scored papers using a previously established scoring tool. The authors provide a brief summary of each article, followed by an analysis of themes based upon their review. The authors identified three main themes: learner characteristics, feedback characteristics, and feedback culture. Within the theme of learner characteristics, the authors discuss the importance of incorporating learner self-assessment by providing benchmarking data and engaging learners in soliciting goal derivation and refinement. The authors note that characteristics of feedback were a dominant finding in their appraisal and that educators should strive to provide high-quality feedback with an emphasis on creating a positive feedback culture, ensuring that feedback is bi-directional, and that it is appropriately timely. They emphasize the importance of faculty development with dedicated feedback training. Finally, the authors discuss the importance of having a positive feedback culture with an expectation of routine feedback.

Relevance to Junior Faculty Members: Junior faculty may struggle with understanding the evidence to support specific feedback practices or newer approaches. This article is a valuable resource for junior faculty because it provides an overview of key themes and some key references within the feedback literature. Readers can utilize this as a launching point for additional reading, as well as to gain an understanding of some of the key emerging themes within the literature. The importance of learner engagement and creating a culture of feedback are particularly important for learner buy-in and long-term adoption and incorporation of feedback recommendations.

Considerations for Faculty Developers: Kornegay and colleagues highlight some of the key literature that can be used to inform faculty development courses for all faculty, regardless of experience. This article can be provided to faculty as pre-reading or utilized as part of the course itself. As noted by the authors, it is important to provide targeted feedback training, as well as ongoing faculty development. Therefore, faculty developers should ensure that feedback training is supported by evidence-based recommendations and incorporates the newer literature. Finally, the increasing data on the value of multi-source feedback should be considered when helping faculty to develop their feedback skills.

*6. *Lefroy J, Watling C, Teunissen P, Brand P: Guidelines: the do’s, don’ts and don’t knows of feedback for clinical education. Perspect Med Educ. 2015, 4:284-299. 10.1007/s40037-015-0231-7 [27]

Summary: This consensus paper from experienced educators summarizes best practices in giving feedback and identifies areas for further scholarly exploration. Although this is not a true systematic review, each recommendation is supported by the authors’ experience, in addition to evidence from the literature. The authors suggest a new definition: “Helpful feedback is a supportive conversation that clarifies the trainee’s awareness of their developing competencies, enhances their self-efficacy for making progress, challenges them to set objectives for improvement, and facilitates their development of strategies to enable that improvement to occur.” They include a summary of characteristics of feedback in the form of “Do’s”, “Don’t” and “Don’t knows” in which they provide 32 guidelines that can be incorporated into clinical practice when giving feedback. These guidelines address the process and content of feedback as well as guidelines that can be applied to create a learning culture conducive to improving the feedback experience for both faculty and learners.

Relevance to Junior Faculty Members: Giving feedback is a difficult task for junior faculty who are transitioning to their new role. Often, they do not have a clear idea of what they should or should not be doing when giving feedback. The article includes an easy-to-read summary table of “Do’s,” “Don’t” and “Don’t knows” for the process and content of feedback. Many of the items on the list of “Do’s” and “Don’ts” within the topic of learning culture can be impacted by junior faculty, such as fostering trusting relationships with learners. This can serve as a quick reference for junior faculty with a review of best practices that they could glance at just before a shift to help remind them of tips for providing helpful feedback. An important point emphasized in the paper is that the impact of feedback is dependent upon proper diagnosis of a learner’s needs and their receptiveness to hearing this feedback, which ultimately reflects on the institution’s learning climate and feedback culture. This paper can serve as a practical tool for junior faculty to help change the climate and feedback culture in their respective institution.

Considerations for Faculty Developers: Faculty developers have an obligation not only to train their faculty how to give helpful feedback, but also to create a learning culture that supports provision and reception of feedback in the clinical environment. This paper provides a refresher on best practices in giving feedback that can be used to structure faculty development sessions. Educational leaders should also follow the guidelines identified by the authors to nurture a supportive learning culture, which can directly impact the effectiveness of feedback. Salient suggestions include prioritizing the role of regular feedback in the institution, promoting direct observation of learners, and providing regular training for faculty to maintain their competencies in giving feedback.

Furthermore, faculty developers can support junior faculty interested in scholarly pursuits in feedback by directing them to help answer the questions identified in the “don’t knows” section.

*7. *Ramani S, Krackov S : Twelve tips for giving feedback effectively in the clinical environment. Med Teach. 2012, 34:787-791. 10.3109/0142159x.2012.684916 [28]

Summary: This article begins with a brief introduction to feedback focused on the shifting view of knowledge acquisition and duration of training towards the achievement of learning outcomes in order to prepare physicians for meeting healthcare needs of the individual and healthcare system. It provides 12 key practical tips that aim to guide the novice in delivering effective feedback, increasing acceptance of feedback, and enabling improvement of performance based upon the feedback. Concrete examples of each tip are provided with illustrations on how to apply them into clinical practice. Some of these 12 tips include establishing a respectful learning environment and basing feedback on direct observation. The article ends with offering suggestions on how to make institutional changes to help create an environment where feedback culture is accepted as the norm.

Relevance to Junior Faculty Members: Delivering effective feedback to learners is an essential, but often daunting, task to many junior faculty members. Its concise delivery of this content in the article makes it an easy resource for the busy junior faculty member. This article provides a useful initial framework for effective feedback delivery by offering straightforward and practical tips that can be easily implemented to improve skills in this domain. 

Considerations for Faculty Developers: Many junior faculty members have little experience in delivering effective feedback. It is essential for faculty developers to provide guidance and teaching in this domain. Although this paper will not make new faculty members feedback experts, it provides an initial, easy-to-follow structured approach for feedback delivery. The final tips in this article are also useful for faculty developers as they are focused on strategies to promote staff development and comfort in delivering feedback providing practical suggestions on how to achieve this.

*8. *Qureshi NS: Giving effective feedback in medical education. Obstet Gynaecol. 2017, 19:243-248. 10.1111/tog.12391 [29]

Summary: This article provides a useful overview of effective feedback delivery by aiming to familiarize the reader with principles of giving effective feedback, models of giving feedback, and an understanding of why medical educators may fail at giving feedback. The author begins with a definition of feedback and relates the importance of feedback to Kolb’s learning cycle, highlighting its importance in supporting learners to reflect. Multiple models for giving structured feedback are discussed with a brief description of each model and its advantages and disadvantages, such as Pendleton’s rule and the trainee-centered model. The article then discusses principles of giving effective feedback in both formal and informal environments with strategies on how to achieve this. The article also explores common mistakes when giving feedback including barriers to giving effective feedback, the ‘feed forward’ concept, embedding feedback in institutional culture, and reflecting on feedback giving skills.

Relevance to Junior Faculty Members: This article provides junior faculty members with a good summary of ways to deliver effective feedback and why it is important. Practical tips, simple feedback strategies, and example questions are also provided, which are helpful for those new to feedback delivery. The article also prompts junior faculty members to reflect on mistakes and barriers to try to avoid in feedback delivery. The ‘feed forward’ approach of looking ahead to future assessments and offering constructive guidance on how to better ensure that assessment has a developmental impact on learning is also well explained.

Considerations for Faculty Developers: This is a valuable article for faculty developers wishing to provide junior faculty members with a concise overview of giving effective feedback. The list of common feedback mistakes, barriers to giving effective feedback, and reasons medical educators fail at feedback are useful considerations for faculty developers wishing to find ways of improving feedback delivery within their department. The article also serves a reminder that embedding feedback into the educational culture of the institution improves its effectiveness while providing helpful suggestions on how to achieve this.

Limitations

This study was not designed to be an exhaustive, systematic review of the literature. Rather, we utilized expert consultation combined with an open social media call to identify a list of the most important articles. This was intentional, so as to identify the most important papers, while maintaining a manageable length for the participants in the study. As such, it is possible that some relevant articles may have been missed. Additionally, while a pure Delphi approach includes only topic experts, we opted to include a combination of experts and junior faculty. By incorporating junior faculty, we are better able to determine articles of interest to this important cohort of medical educators [10].

## Conclusions

We present eight key papers addressing the topic of feedback with discussions and applications for junior faculty members and those leading faculty development initiatives. These papers provide a basis on which junior faculty members might build for designing the methods by which they can appraise their local programs and projects. These articles are applicable to physicians at all career stages and can help inform them on how to become more experienced with giving feedback. 
